# Local excision versus radical surgery for anal squamous cell carcinoma: a multicenter study in Japan

**DOI:** 10.1007/s10147-024-02498-z

**Published:** 2024-03-25

**Authors:** Shin Murai, Hiroaki Nozawa, Kazutaka Yamada, Yasumitsu Saiki, Kazuhito Sasaki, Koji Murono, Shigenobu Emoto, Hiroyuki Matsuzaki, Yuichiro Yokoyama, Shinya Abe, Yuzo Nagai, Yuichiro Yoshioka, Takahide Shinagawa, Hirofumi Sonoda, Kenichi Sugihara, Yoichi Ajioka, Soichiro Ishihara

**Affiliations:** 1 Department of Surgical Oncology, Faculty of Medicine, The University of Tokyo, 7-3-1 Hongo, Bunkyo-Ku, Tokyo, 113-0033 Japan; 2 Department of Surgery, Takano Hospital, Kumamoto, Japan; 3 Tokyo Medical and Dental University, Tokyo, Japan; 4 Division of Molecular and Diagnostic Pathology, Graduate School of Medical and Dental Sciences, Niigata University, Niigata, Japan; 5 Project Committee for Handling Anal Cancers, The Japanese Society for Cancer of the Colon and Rectum, Tokyo, Japan

**Keywords:** Anal cancer, Local excision, Radical surgery

## Abstract

**Background:**

The standard treatment for anal squamous cell carcinoma is chemoradiation therapy (CRT), but there is a possibility of over-treatment for early-stage disease. cTisN0 and cT1N0 disease is currently indicated for local excision, but it is unclear whether the indication of local excision can be expanded to cT2N0 disease.

**Methods:**

126 patients with cTis-T2N0 anal cancer treated at 47 centers in Japan between 1991 and 2015 were included. Patients were first classified into the CRT group and surgical therapy group according to the initial therapy, and the latter was further divided into local excision (LE) and radical surgery (RS) groups. We compared prognoses among the groups, and analyzed risk factors for recurrence after local excision.

**Results:**

The CRT group (*n* = 87) and surgical therapy group (*n* = 39) showed no difference in relapse-free survival (*p* = 0.29) and overall survival (*p* = 0.94). Relapse-free survival curves in the LE (*n* = 23) and RS groups (*n* = 16) overlapped for the initial 3 years, but the curve for the LE group went lower beyond (*p* = 0.33). By contrast, there was no difference in overall survival between the two groups (*p* = 0.98). In the LE group, the majority of recurrences distributed in locoregional areas, which could be managed by salvage treatments. Muscular invasion was associated with recurrence after local excision (hazard ratio: 22.91, *p* = 0.011).

**Conclusion:**

LE may be applied to selected patients with anal cancer of cTis-T2N0 stage. Given the high risk of recurrence in cases with muscular invasion, it may be important to consider close surveillance and additional treatment in such patients.

## Introduction

Anal squamous cell cancer is an uncommon tumor that represents 4% of all cancers in the lower gastrointestinal tract [1]. In 1970s, radical surgery such as abdominoperineal resection (APR) was the mainstay of treatment for anal cancer. In 1980s, Nigro et al. performed chemoradiation therapy (CRT) for anal cancer which provided good prognosis and obviated permanent stoma in advanced stage patients. Based on the results, there was a paradigm shift from surgical therapy to CRT in the treatment of anal cancer [2, 3]. However, no studies directly compared radical surgery and CRT. Moreover, there are several concerns in implementing CRT. Late complications related to CRT can be problematic, including chronic diarrhea, fecal incontinence, and sexual dysfunction [4, 5]. Additionally, CRT is a risk for secondary carcinogenesis [6]. Therefore, there is an argument that CRT may be over-treatment for early-stage anal cancer [7]. In fact, satisfactory long-term outcomes of local excision for cT1N0 anal cancer were recently demonstrated [8–12].

Regarding cT2N0 anal cancers, only a couple of studies investigated the outcomes of local excision. One study concluded that local excision in cT2 tumors is not recommended due to insufficient margins [13], whereas the other argued that local excision is acceptable for cT1–2 cases because postoperative radiation therapy (RT)/CRT improves the prognosis [14]. These outcomes of local excision were not compared to those of other treatment strategies in both studies.

In this study, we first addressed whether surgical therapy and CRT provide comparable prognosis for early-stage anal cancer including cTis-2N0 cases. Then, by confining the study subject to patients receiving surgical therapy, we compared outcomes of radical surgery and local excision to determine whether the latter treatment is feasible and pragmatic.

## Methods

### Patients

A total of 436 patients with anal canal squamous cell carcinoma were treated at 47 tertiary centers in Japan (listed in Acknowledgements) between 1991 and 2015. Among these, 126 patients with cTis-2N0 anal canal squamous cell carcinoma were enrolled in this study. Disease was staged according to the UICC TNM classification (8th edition) [15]. Patients were first classified into the CRT group and ‘Surgical therapy’ group according to the initial therapy, and the latter was further divided into ‘local excision (LE)’ and ‘radical surgery (RS)’ groups.

Consent to conduct the research was provided by the JSCCR ethical committee.

### Outcome measurements

Clinical parameters including sex and age, clinical T stage, and pathological differentiation of tumor were compared between the CRT group and surgical therapy group.

The same clinical parameters and pathological findings such as histological type, pathological T and N stages, lymphocytic invasion and venous invasion, and resection margin were compared between the LE group and RS group. Recurrence types were classified into locoregional recurrence and distant metastasis. Locoregional recurrence included pelvic, perineal, and inguinal lymph nodal recurrence. Distant metastasis was defined as recurrence in organs other than the local. Relapse-free survival (RFS) was defined as time between the date of surgery and relapse. Overall survival (OS) was defined as time between the starting date of initial therapy and death from any causes. RFS and OS were compared between the CRT group and Surgical therapy group, and also compared between the LE group and RS group. Surgical complications of Clavien–Dindo classification [16] grade 2 or higher were addressed for the LE group, while adverse events of the National Cancer Institute’s Common Terminology Criteria for Adverse Events (CTCAE) v5.0 [17] grade 2 or higher were reviewed for the CRT group.

### Statistical analysis

An un-paired t test was used to compare continuous variables and the Yates’ correction or Fisher’s exact test was used to compare categorical data. RFS rate was estimated using the Kaplan–Meier method and compared with the log-rank test. Propensity score matching was used to minimize the selection bias due to unbalanced baseline characteristics in cT2 tumors between the CRT and LE groups. The parameters that were different between the two groups and other potential factors that could influence OS including sex, age, histology were selected to create a propensity score ranging from 0 to 1 using a logistic regression model. Then, a one-to-one match between the CRT and LE groups was performed using nearest-neighbor matching with a caliper width equal to 0.2 of the standard deviation of the logit of the propensity score. All analyses were performed with the JMP 15.0 software program (SAS Institute, Inc., Cary, NC, USA), and differences with a *p* < 0.05 were considered significant.

## Results

### Patient characteristics

**Fig. 1:**
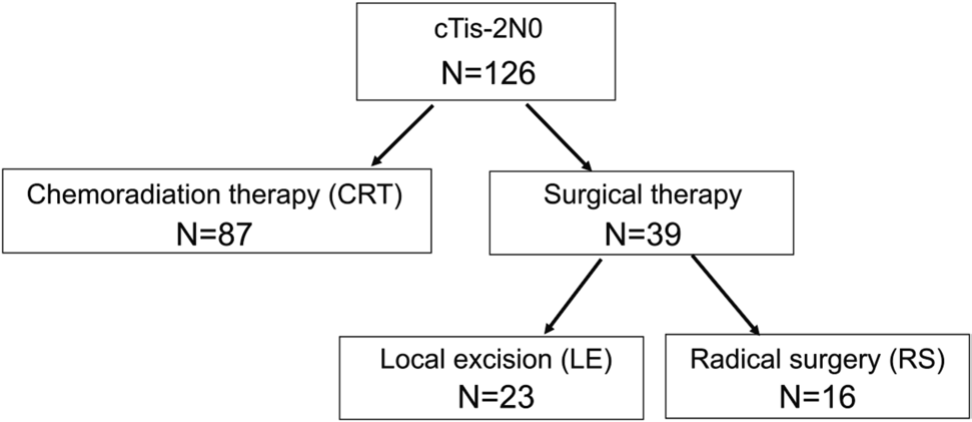
Flow diagram of the study cohort. Patients were first classified into the chemoradiation therapy (CRT) and surgical therapy groups according to the initial therapy, and the latter was further divided into local excision (LE) and radical surgery (RS) groups

Among 126 patients with cTis-2N0 anal canal squamous cell carcinoma enrolled in this study, 87 and 39 patients were classified to the CRT group and surgical therapy group, respectively. Moreover, 23 patients were classified to the LE group, and 16 patients to the RS group (Fig. 1).

### Comparison of the CRT and surgical therapy groups

Background characteristics in the CRT group and surgical therapy group are shown in Table 1. Clinical T stage in the CRT group was more advanced than in the surgical therapy group (*p* = 0.008). There was no significant difference in other parameters.

**Table 1:** Patient demographics and tumor characteristics in CRT and surgical therapy groups

	CRT	Surgical therapy	*p*-value
Number of patients	87	39	
Sex			
Male	24 (28%)	14 (36%)	0.35
Age, year			
Median	65	67	0.42
cT stage			
cTis	1 (1%)	6 (15%)	0.008
cT1	37 (43%)	14 (36%)	
cT2	49 (56%)	19 (49%)	
Histology			
Well or mod	30 (34%)	18 (46%)	0.38
Others	13 (15%)	13 (33%)	
Unknown	44 (51%)	9 (21%)	

*CRT* chemoradiation therapy, *well* well differentiated, *mod* moderately differentiated, *others* poorly differentiated, adenosquamous, or basaloid

**Fig. 2:**
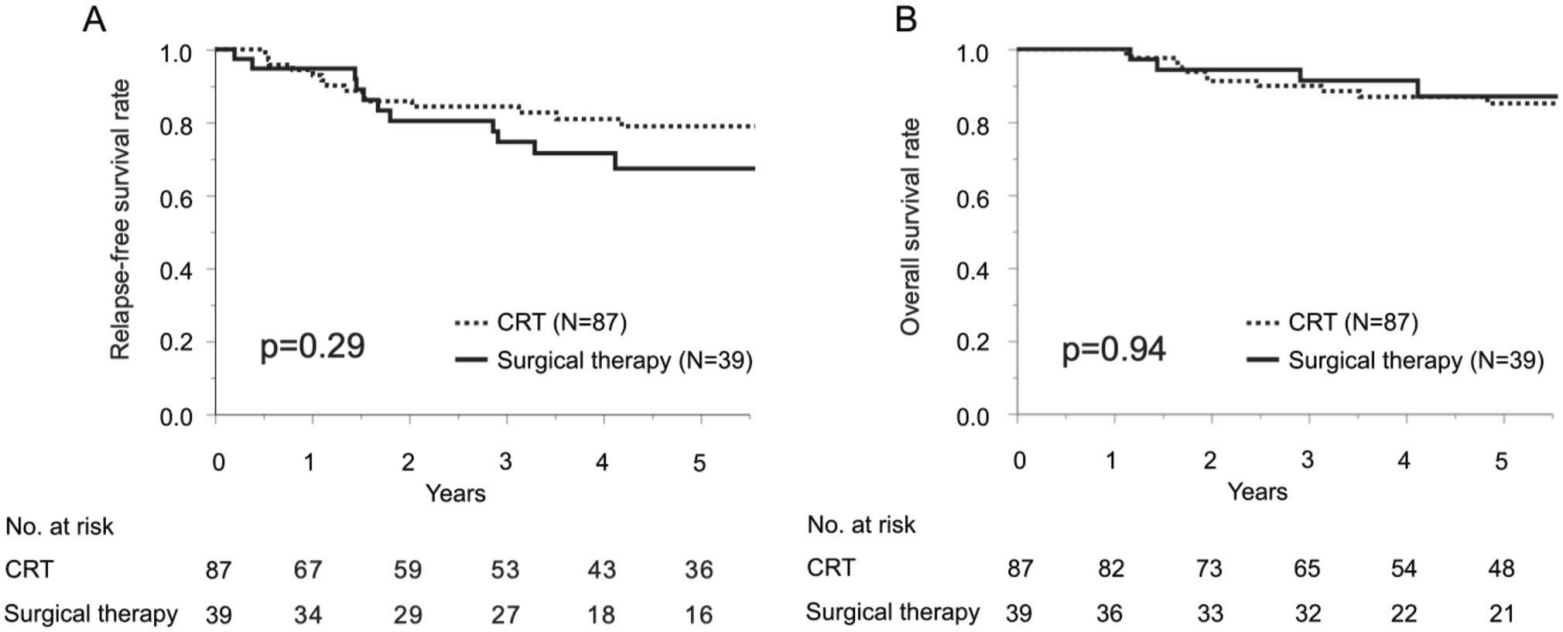
**A** Relapse-free survival (RFS) in patients with cTis-2N0 anal squamous carcinoma. Bold line indicates RFS curve for surgical therapy group, and dashed line indicates RFS curve for CRT group. **B** Overall survival (OS) in patients with cTis-2N0 anal squamous carcinoma. Bold line indicates OS curve for surgical therapy group, and dashed line indicates OS curve for CRT group

There was no significant difference in RFS between the CRT group and surgical therapy group (*p* = 0.29). The 5-year RFS was 79% for the CRT group and 67% for the surgical therapy group (Fig. 2). In addition, OS was comparable between the CRT group and surgical therapy group (*p* = 0.94). The 5-year OS was 85% for the CRT group and 87% for the surgical therapy group (Fig. 2).

In the LE group, there were no short-term complications of Clavien–Dindo classification grade 2 or higher. Only one female patient (4%) who underwent LE with positive resection margin followed by adjuvant CRT developed anal stenosis 5 years later. On the other hand, 41 of 87 patients (47%) of the CRT group had CTCAE grade 2 or higher adverse events (Table 2).

**Table 2:** Treatment-related adverse events in 87 patients of CRT group according to CTCAE v5.0

Adverse event	≧ Grade 2	Grade 2	Grade 3	Grade 4	Grade 5
Any	41 (47%)	40 (46%)	21 (24%)	2 (2.3%)	1 (1.1%)
Fatigue	1 (1.1%)	1 (1.1%)			
Anorexia	2 (2.3%)	2 (2.3%)			
Nausea	3 (3.4%)	2 (2.3%)	1 (1.1%)		
Diarrhea	6 (6.9%)	6 (6.9%)			
Dermatitis	19 (22%)	12 (14%)	6 (6.9%)		
Stomatitis	2 (2.3%)	2 (2.3%)			
Proctitis	1 (1.1%)	1 (1.1%)			
Small intestine perforation	1 (1.1%)				1 (1.1%)
Anal pain	4 (4.6%)	4 (4.6%)			
Fecal incontinence	1 (1.1%)		1 (1.1%)		
Urinary disorder	1 (1.1%)	1 (1.1%)			
Urethral stricture	1 (1.1%)	1 (1.1%)			
Anemia	2 (2.3%)	2 (2.3%)			
Leukopenia	7 (8%)	3 (3.4%)	3 (3.4%)	1 (1.1%)	
Neutropenia	7 (8%)	1 (1.1%)	5 (5.7%)	1 (1.1%)	
Febrile neutropenia	2 (2.3%)		2 (2.3%)		
Thrombocytopenia	4 (4.6%)	1 (1.1%)	3 (3.4%)		
Liver dysfunction	1 (1.1%)	1 (1.1%)			

*CRT* chemoradiation therapy

### Comparison of the LE and RS groups

Patient characteristics in the LE group and RS group are shown in Table 3. Clinical T stage in the RS group were more advanced than in the LE (*p* = 0.01). On the other hands, the LE group showed a broader range of pT stage than the RS group (*p* = 0.003). Approximately 20% of the RS group showed regional node metastasis. More patients (74%) in the RS group showed muscular invasion compared to the LE group (18%, *p* = 0.0001). Five patients in the LE group had a positive resection margin; all underwent adjuvant therapy (ablation in 1, RT in 1, and CRT in 3 patients) and subsequently had no recurrence.

**Table 3:** Patient demographics and tumor characteristics in local excision and radical surgery groups

	Local excision	Radical surgery	*p*-value
Number of patients	23	16	
Sex			
Male	10 (43%)	4 (25%)	0.23
Age, year			
Median	68	65	0.38
Location			
Anal canal	17 (74%)	15 (93%)	0.16
Anal margin	5 (26%)	1 (7%)	
cT stage			
cTis	6 (26%)	0 (0%)	0.013
cT1	9 (35%)	5 (31%)	
cT2	8 (39%)	11 (69%)	
pT stage			
pTis	5 (22%)	0 (0%)	0.031
pT1	8 (35%)	4 (25%)	
pT2	9 (39%)	12 (75%)	
pT3	1 (4%)	0 (0%)	
pT4	0 (0%)	0 (0%)	
pN stage			
pN0	0 (0%)	7 (44%)	N/E
pN1	0 (0%)	3 (19%)	
pNX	23 (100%)	6 (37%)	
Histology			
Well or mod	9 (39%)	9 (56%)	0.65
Others	5 (22%)	7 (44%)	
Unknown	9 (39%)	0 (0%)	
Lymphatic invasion			
Absent	14 (61%)	8 (50%)	0.50
Vascular invasion			
Absent	16 (70%)	7 (44%)	0.11
Muscular invasion			
Absent	17 (74%)	3 (18%)	0.0001
Resection margin			
Positive	5 (22%)	0 (0%)	0.022
Negative	14 (61%)	15 (94%)	
Unknown	4 (17%)	1 (6%)	
Adjuvant therapy			
Yes	6 (26%)	2 (13%)	0.30

*N/E* not evaluated, *well* well differentiated, *mod* moderately differentiated, *others* poorly differentiated, adenosquamous, or basaloid

**Fig. 3:**
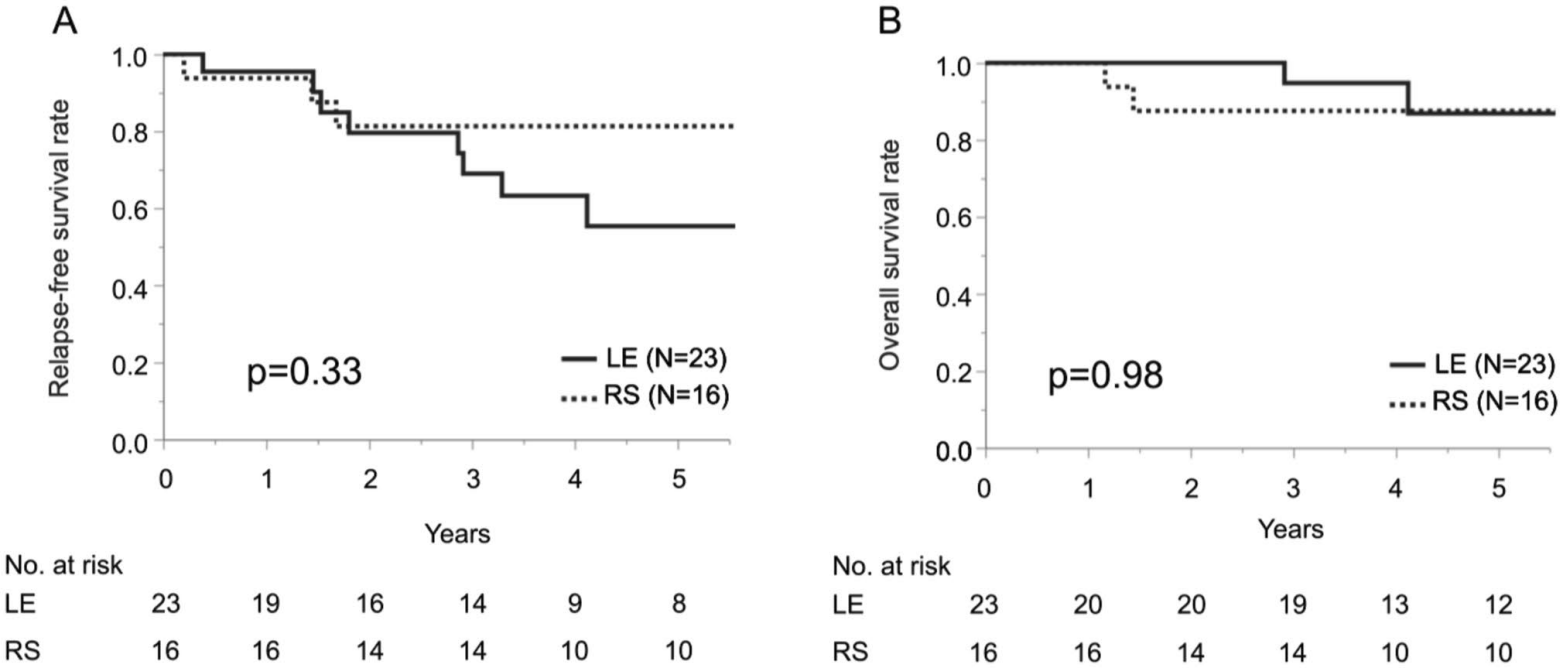
**A** Relapse-free survival (RFS) in patients with cTis-2N0 anal squamous carcinoma. Bold line indicates RFS curve for local excision (LE) group, and dashed line indicates RFS curve for radical surgery (RS) group. **B** Overall survival (OS) in patients with cTis-2N0 anal squamous carcinoma. Bold line indicates OS curve for local excision (LE) group, and dashed line indicates OS curve for radical surgery (RS) group

RFS curves in the LE and RS groups overlapped for the initial 3 years, but the curve for the LE group went lower beyond (*p* = 0.33). The 5-year RFS rate was 55.3% for the LE group and 71.9% for the RS group. By contrast, there was no significant difference in OS between the two groups (*p* = 0.98). The 5-year OS rate was 87% for the LE group and 88% for the RS group (Fig. 3).

### Recurrence pattern in the LE and RS groups

Seven patients (30%) relapsed in the LE group with the median follow-up of 40 months, whereas three patients (19%) relapsed in the RS group (median follow-up: 72 months, Table 4). Recurrence rate after LE was 27% for cTis-1 disease (4 of 15 patients) and 38% for cT2 disease (3 of 8 patients, *p* = 0.66). All 10 patients with recurrence in both groups had primary cancer of the anal canal. One patient in each group developed distant metastasis. A male patient in the LE group had recurrence in para-aortic lymph nodes, and received radiotherapy. A female patient in the RS group had liver metastasis, and moved to another hospital.

**Table 4:** Recurrence in surgical therapy group

	Local excision (*n* = 23)	Radical surgery (*n* = 16)
No. of patients with recurrence	7 (30%)	3 (19%)
Recurrence site		
Locoregional		
Pelvic/perineal	4 (17%)	2 (13%)
Inguinal lymph nodes	2 (9%)	0 (0%)
Distant		
Para-aortic lymph nodes	1 (4%)	0 (0%)
Liver	0 (0%)	1 (6%)
Lung	0 (0%)	0 (0%)
Follow up, months		
Median	40	72

In the LE group, two patients with inguinal nodal metastases underwent nodal dissection. Locoregional recurrence was treated by rectal amputation in two patients and CRT in one patient. One female patient with locoregional recurrence in LE group was advised to receive CRT, but she preferred to be followed with palliative care. In the RS group, one patient with iliac nodal metastases underwent radiation therapy, and another with perineal recurrence received additional local excision followed by radiation therapy.

### Recurrence risk in the LE group

We investigated factors associated with recurrence in patients of the LE groups. The presence of muscular invasion was the only risk factor for recurrence (hazard ratio: 22.91, 95% confidence interval: 2.05–256.6, *p* = 0.011, Table 5). Other parameter had no significant associations with recurrence.

**Table 5:** Recurrence risk in local excision group

	Univariate analysis		
	HR	95% CI	*p*-value
Sex			
Male vs female	0.86	0.20–3.61	0.83
cT			
cTis-1 vs cT2	0.57	0.14–2.27	0.42
pT			
pTis-1 vs pT2	0.75	0.19–3.04	0.69
Histology			
Well or mod vs Others	0.48	0.12–1.95	0.31
Lymphatic invasion			
Present vs Absent	1.19	0.28–4.97	0.82
Vascular invasion			
Present vs Absent	0.79	0.16–3.97	0.78
Muscular invasio			
Present vs Absent	22.91	2.05–256.6	0.011
Resection margin			
Positive vs Negative	4.27	0.5–36.3	0.18
Adjuvant therapy			
Yes vs No	0.59	0.12–2.95	0.50

*HR* hazard ratio, *CI* confidence interval, *well* well differentiated, *mod* moderately differentiated, *others* poorly differentiated, adenosquamous, or basaloid

### Comparison of cT2 tumors in the CRT and LE groups

**Fig. 4:**
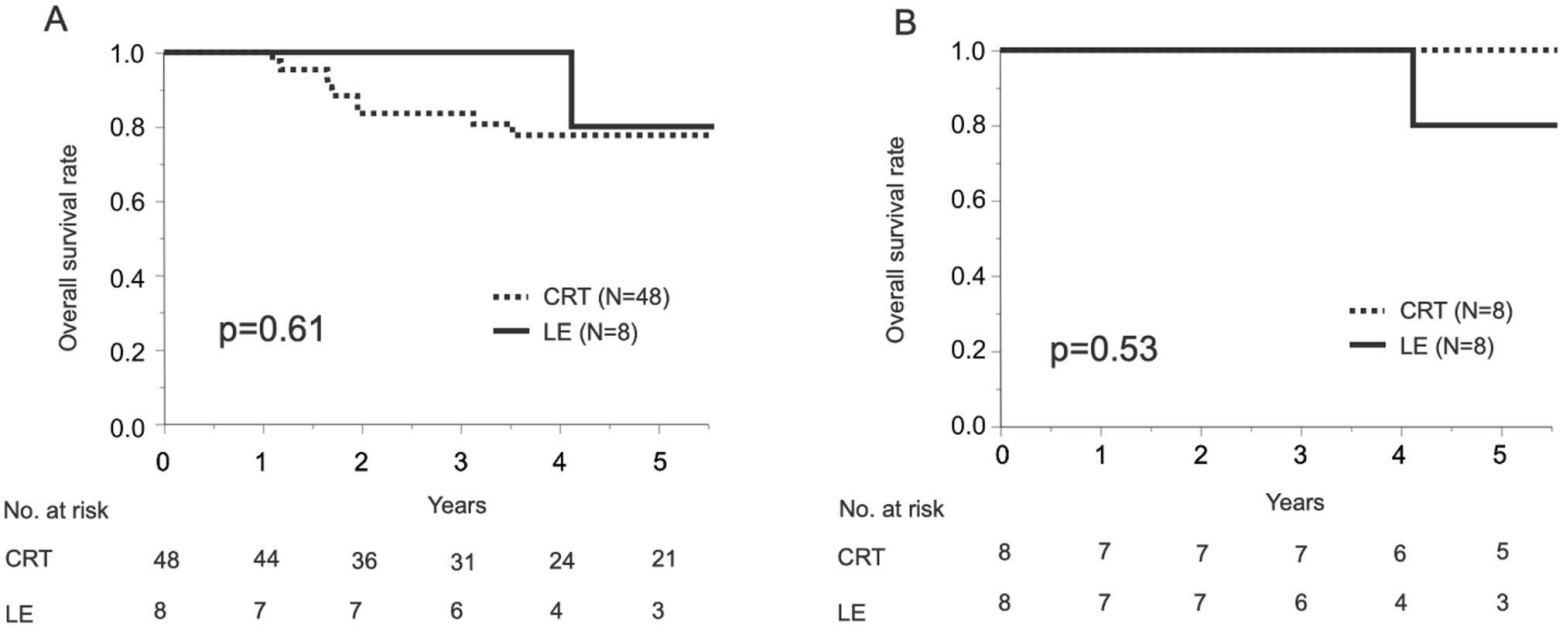
Overall survival (OS) in patients with cT2N0 anal squamous carcinoma. Bold line indicates OS curve for local excision (LE) group, and dashed line indicates OS curve for CRT group. **A** Before propensity score matching. **B** After propensity score matching

Finally, the prognosis of cT2 tumors was compared between the CRT and LE groups. The clinical and pathological data of patients in the CRT and LE groups were summarized in Table 6. Before propensity score matching, there was a difference in the proportion of various histologies. We applied a propensity score matching method to create 8 matched pairs from the original cohort to adjust for the bias for survival analyses (Table 6). There was no significant difference in OS between the CRT and LE groups both before and after matching (Fig. 4).

**Table 6:** Patient demographics and tumor characteristics of cT2 tumors in CRT and Local excision groups

Variable	Before propensity score matching			After propensity score matching		
	CRT (*n* = 48)	Local excision (*n* = 8)	*p*-value	CRT (*n* = 8)	Local excision (*n* = 8)	*p*-value
Sex						
Male	13 (27%)	2 (25%)	0.93	1 (12%)	2 (25%)	0.52
Age, year						
Median	65	72	0.41	72	72	0.67
Histology						
Well or mod	14 (29%)	6 (75%)	0.003	5 (62%)	6 (75%)	0.59
Others	8 (17%)	2 (25%)		3 (38%)	2 (25%)	
Unknown	27 (57%)	0		0	0	

*CRT* chemoradiation therapy, *well* well differentiated, *mod* moderately differentiated, *others* poorly differentiated, adenosquamous, or basaloid

## Discussion

There were a limited number of studies that investigated the outcomes between CRT and surgical treatment for anal squamous cell cancer; regarding cT1 disease, there was accumulating evidence that local excision is comparable to CRT in terms of prognosis [8]. However, for tumors of the anal canal origins, there is a concern that an adequate margin of dissection may not be obtained [13, 14]. The National Comprehensive Cancer Network guidelines recommend local excision only for cT1N0 lesions localized in the anal margin [18]. On the other hand, only a few reports investigated the efficacy of local excision for cT2N0 anal cancer [13, 14]. However, outcomes of CRT vs surgical treatment were not directly compared in the literature including the above studies [13, 14]. In our study, there was no difference in RFS and OS between CRT and surgical therapy for cTis-2N0 anal cancer (Fig. 2). No difference in OS was observed between the treatment groups even in the limited cohort with cT2N0 cancer (Fig. 4). Thus, both CRT and surgical therapy may be recommended in patients with anal cancer of these early stages. Moreover, LE seemed less invasive than CRT, since no perioperative complications related to LE were observed, whereas nearly half of the CRT group experienced CTCAE grade 2 or severer adverse events.

No previous reports compared the prognosis of LE and RS. Patients in the LE and RS groups showed comparable RFS for first three years, but the LE group was more likely to develop recurrence thereafter in our study (Fig. 3). The majority of patients in the LE group (86%) had locoregional recurrence (Table 4). Moreover, it was considered that all locoregional recurrent lesions could be potentially managed by CRT or salvage surgery in the LE group. Despite of the discrimination of RFS curve beyond 3 years after surgery in the LE and RS groups, OS curves of both groups were almost identical. The results suggest that effective salvage treatment contributed to a good prognosis even in recurrent cases in the LE group. To prescribe salvage therapy in a timely manner,

close surveillance may be necessary for a long period in the LE group.

Maccabe et al. reported that R1 resection rate in local excision was 54% for tumors of the anal margin and 93% for tumors of the anal canal [13]. They also reported that there was no difference in recurrence rate between the anal margin and anal canal origins, as adjuvant therapy was usually performed in cases with inadequate resection margin [13]. Similarly, Leon et al. also reported that postoperative RT/CRT after local excision improved OS and disease-free survival in patients who underwent local excision with positive resection margin [14]. These results suggested that recurrence in anal cancer may be manageable if appropriate adjuvant therapy is performed. In line with these previous reports, recurrence was evaded by the administration of adjuvant therapy in all cases with positive resection margin of the LE group in this study.

Regarding the relationship between pathological findings and prognosis after local excision, Maccabe et al. reported that anal cancer with lymphovascular invasion carried a high risk for recurrence, but muscular invasion was not examined [13]. On the other hand, we demonstrated that muscular invasion was a risk factor for recurrence in anal cancer (Table 5). Alana et al. reported that anal superficially invasive squamous cell carcinoma (SISCCA), defined as an invasive lesion within 3 mm vertically and 7 mm horizontally, showed a good prognosis when treated by local excision [19]. However, it is difficult to make a correct diagnosis of SISCCA preoperatively. In contrast, muscular infiltration of the tumor may be evaluated preoperatively with endoscopic ultrasound and magnetic resonance imaging [20, 21].

This study has several limitations. First, this study is a retrospective study, examining a small number of patients. Especially, the similar prognosis of cT2N0 cancer between the CRT and LE groups, should be evaluated with caution because the very small number of cases might result in a type II error. Second, the initial treatment at diagnosis and treatment at the time of recurrence depended on the discretion at each hospital due to a multi-institutional study. Lastly, we did not investigate anal function and quality of life after surgery, and did not assess patients' satisfaction with the treatment.

Our study showed that local excision may be a possible treatment option for cTis-2N0 anal squamous cell carcinoma, as OS in the LE group was comparable to that in the RS group probably owing to salvage therapy for locoregional lesions that accounted for the majority of recurrent cases. Given the high risk of recurrence in cases showing muscular invasion, it may be important to perform close surveillance and to consider additional treatment in such patients.

## Data Availability

The data that support the findings of this study are available from the corresponding author upon reasonable request.
